# Effect of cAMP signaling on expression of glucocorticoid receptor, Bim and Bad in glucocorticoid-sensitive and resistant leukemic and multiple myeloma cells

**DOI:** 10.3389/fphar.2015.00230

**Published:** 2015-10-13

**Authors:** Hongli Dong, Michael E. Carlton, Adam Lerner, Paul M. Epstein

**Affiliations:** ^1^Department of Cell Biology, University of Connecticut Health Center, FarmingtonCT, USA; ^2^Section of Hematology and Oncology, Evans Department of Medicine, Boston Medical Center, BostonMA, USA

**Keywords:** cAMP signaling, glucocorticoid signaling, Bim, Bad, leukemia, multiple myeloma, glucocorticoid receptors, phosphodiesterases

## Abstract

Stimulation of cAMP signaling induces apoptosis in glucocorticoid-sensitive and resistant CEM leukemic and MM.1 multiple myeloma cell lines, and this effect is enhanced by dexamethasone in both glucocorticoid-sensitive cell types and in glucocorticoid-resistant CEM cells. Expression of the mRNA for the glucocorticoid receptor alpha (GR) promoters 1A3, 1B and 1C, expression of mRNA and protein for GR, and the BH3-only proapoptotic proteins, Bim and Bad, and the phosphorylation state of Bad were examined following stimulation of the cAMP and glucocorticoid signaling pathways. Expression levels of GR promoters were increased by cAMP and glucocorticoid signaling, but GR protein expression was little changed in CEM and decreased in MM.1 cells. Stimulation of these two signaling pathways induced Bim in CEM cells, induced Bad in MM.1 cells, and activated Bad, as indicated by its dephosphorylation on ser112, in both cell types. This study shows that leukemic and multiple myeloma cells, including those resistant to glucocorticoids, can be induced to undergo apoptosis by stimulating the cAMP signaling pathway, with enhancement by glucocorticoids, and the mechanism by which this occurs may be related to changes in Bim and Bad expression, and in all cases, to activation of Bad.

## Introduction

Glucocorticoids remain a central component of all therapeutic regimens used to treat leukemia and multiple myeloma (Ploner et al., 2005; Sionov et al., 2008; Bhadri et al., 2012). However, about 20% of patients demonstrate resistance to glucocorticoids and do not respond to treatment, and invariably, of those that do respond, many develop resistance to glucocorticoids later during treatment, causing them to relapse, with a very poor prognosis (Haarman et al., 2003; Frankfurt and Rosen, 2004; Schmidt et al., 2004; Ploner et al., 2005; Sionov et al., 2008; Bhadri et al., 2012). The molecular basis of glucocorticoid resistance is not fully understood. A number of studies using *in vitro* model systems have suggested that it may be associated with a decrease in the expression or alteration of the glucocorticoid receptor (GR), such that the steps normally carried out by the GR that lead to therapeutic benefit are muted (Moalli and Rosen, 1994; Gaynon and Carrel, 1999; Schmidt et al., 2004, 2006; Ploner et al., 2005); however, at least one study finds no correlation with GR expression or function, but finds instead a correlation with the profound attenuation of the induction of the BH3-only pro-apoptotic protein, Bim (Bachmann et al., 2005). Studies using acute lymphocytic leukemic (ALL) cells obtained from patients, as well as 15 T-ALL cell lines grown directly from patients’ cells without prior drug exposure in culture, also indicated that resistance could not be attributed to mutations in GR or variations in levels of its expression (Tissing et al., 2006; Bachmann et al., 2007; Beesley et al., 2009). We found that stimulation of the cAMP signaling pathway can overcome glucocorticoid resistance in chronic lymphocytic leukemia (CLL) cells, and in the ALL cell line, CCRF-CEM (Tiwari et al., 2005; Lerner and Epstein, 2006; Dong et al., 2010). The mechanism by which this synergistic effect between stimulation of the cAMP and glucocorticoid signaling pathways occurs, to induce apoptosis of glucocorticoid resistant cells, is, however, still not fully understood. The purpose of this study is to examine the mechanism(s) by which cAMP and glucocorticoid signaling synergize to induce apoptosis of leukemic and multiple myeloma cells.

With respect to leukemia, it appears that the BH3-only pro-apoptotic proteins, Bim and Bad, may be very critical regulators of apoptosis of these cells. In a DNA microarray analysis to uncover genes important in glucocorticoid-induced apoptosis of leukemic cells, Bim was identified as one of the proteins whose expression was most upregulated (Wang et al., 2003). Additionally, studies with mice made deficient for the production of Bim indicate that Bim plays a key role in mediating apoptosis of B and T lymphocytes (Hildeman et al., 2002; Mouhamad et al., 2004). And silencing of the Bim gene with RNAi inhibits glucocorticoid-induced apoptosis of leukemic cells (Abrams et al., 2004). Bim exists as three alternate spliced forms, a short form, BimS, a long form, BimL, and an extra long form, BimEL. Both the turnover and activation of BimEL have been shown to be regulated by its phosphorylation by the MAP Kinases, ERK 1/2 (Ley et al., 2004) and JNK (Putcha et al., 2003). Additionally, studies have shown that the expression of Bim at the gene level is under direct control of the Forkhead transcription factor, FOXO (FKHR; Dijkers et al., 2000). FOXO itself can be phosphorylated and inhibited by the growth promoting kinase PKB/Akt (Burgering and Medema, 2003). PKB/Akt was shown to be inhibited in lymphoma cells by stimulating the cAMP pathway with phosphodiesterase4 (PDE4) inhibitors (Smith et al., 2005), and a similar effect was also seen in mouse embryo fibroblasts (Kuiperij et al., 2005). Hence, stimulating the cAMP pathway and inhibiting PKB/Akt, would be expected to disinhibit FOXO and drive the expression of Bim. And indeed, it was shown that stimulation of the cAMP and glucocorticoid pathways in mouse S49 lymphoma and human CCRF-CEM leukemia cells resulted in a synergistic increase in the expression of Bim (Zhang and Insel, 2004). Bad also appears to be a key player in the regulation of lymphoid cell apoptosis. The activity of Bad is largely controlled by its state of phosphorylation. Studies with interleukin-3 dependent lymphoid cells have shown that when Bad is phosphorylated it is sequestered into an inactive complex with the chaperone protein, 14-3-3. Upon stimulation of its dephosphorylation, it dissociates from 14-3-3 and can then act to initiate apoptosis (Chiang et al., 2001). In cells from patients with CLL, stimulation of the cAMP pathway with the PDE4 inhibitor rolipram led to activation of the protein phosphatase 2A, which dephosphorylated Bad, on its ser112 residue, resulting in increased apoptosis of the leukemic cells (Moon and Lerner, 2003).

We showed previously that inhibitors of PDE4 induce apoptosis of primary CLL cells and synergize with glucocorticoids in doing so (Tiwari et al., 2005). Using glucocorticoid-sensitive and resistant CCRF-CEM leukemic cell lines that we established previously (Tiwari et al., 2005), and established glucocorticoid-sensitive and resistant multiple myeloma cell lines (Greenstein et al., 2003), we examined the effects of stimulation of these two signaling pathways on these glucocorticoid-sensitive and resistant cell types, in relation to their effects on the expression of the mRNA for the GR alpha promoters 1A3, 1B and 1C, expression of mRNA and protein for GR, and the BH3-only proapoptotic proteins, Bim and Bad, and the phosphorylation state of Bad, as a means to examine the mechanism for the synergy between stimulation of the cAMP and glucocorticoid signaling pathways on apoptosis of these cells.

## Materials and Methods

### Materials

Dexamethasone, hydrocortisone, rolipram, forskolin, and 1,9-dideoxyforskolin were obtained from Biomol (Plymouth Meeting, PA, USA). Phenazine methosulfate (PMS) and protease inhibitor cocktail for use with mammalian cell and tissue extracts were from Sigma-Aldrich (St. Louis, MO, USA). [3-(4,5-dimethylthiazol-2-yl)-5-(3-carboxymethoxyphenyl)-2-(4-sulfophenyl)-2H-tetrazolium, inner salt] (MTS) was from Promega (Madison, WI, USA). Primary rabbit polyclonal antibodies directed against GR, Bim, Bad, and S112 phospho-Bad were obtained either from Santa Cruz Biotechnology (Santa Cruz, CA, USA), Cell Signaling Technologies (Danvers, MA, USA), Biosource International (Camarillo, CA, USA), or Zymed Laboratories (South San Francisco, CA, USA). The specific antibodies used were: GR, Santa Cruz sc-8992; Bim, Santa Cruz sc-11425 and Zymed 38-6500; Bad, Santa Cruz sc-7869 and Cell Signaling Technologies 9292; phospho-Bad, Santa Cruz sc-7998-R, Cell Signaling Technologies 9291 and Biosource International 44-522. Primary rabbit monoclonal antibody directed against glyceraldehyde-3-phosphate dehydrogenase (GAPDH) was obtained from Cell Signaling Technologies. Anti-Rabbit IgG-horseradish peroxidase was obtained from GE Healthcare (Piscataway, NJ, USA).

### Cell Culture

Glucocorticoid-sensitive (CEM-S2) and glucocorticoid-resistant (CEM-R8) CCRF-CEM T leukemic cell lines were isolated as described previously (Tiwari et al., 2005). Established glucocorticoid-sensitive (MM.1S) and glucocorticoid-resistant (MM.1R) cell lines (Greenstein et al., 2003) were also obtained for this study. CEM and MM.1 cells were maintained in RPMI 1640 medium supplemented with 10% fetal bovine serum, 2 mM L-glutamine, 100 U/ml penicillin, and 100 μg/ml streptomycin (all from Invitrogen, Carlsbad, CA, USA), at 37°C in a humidified atmosphere of 95% air and 5% CO_2_.

### MTS Assay

MTS assays were conducted as described previously (Dong et al., 2010, 2015). CEM and MM.1 cells were plated in triplicate at a density of 3 × 10^4^ cells/well in 96-well flat-bottom tissue culture plates in RPMI 1640 medium supplemented with 10% heat-inactivated fetal bovine serum, 2 mM L-glutamine, 100 U/ml penicillin, 100 μg/ ml streptomycin, in a total volume of 0.1 ml of fresh medium containing the test reagents or vehicle as indicated. Following incubation at 37°C for 72 h, 20 ul of a combined solution of MTS (2 mg/ml)/PMS (0.92 mg/ml; 20:1, mixed immediately before use) was added to each well, and the plates incubated for an additional 2 h at 37°C, protected from light, following which the absorbency (OD) of the formazan product formed was determined at 492 nm using a microtiter plate reader (Titertek Multiscan Plus model MK II from Labsystems). The assay was optimized by seeding different amounts of cells at zero time, and determining changes in cell number over 24, 48, and 72 h times to establish ranges of linearity. All assays were done under conditions that maintained them in these linear ranges. With the exception of dexamethasone, all reagents tested were dissolved in DMSO and diluted into the cell culture medium such that the final concentration of DMSO in the assay was 0.1%. Percent cell viability is proportional to the amount of formazan product formed and was calculated as follows: (OD test sample – OD blank)/(OD control – OD blank) × 100, where blank refers to plate wells where media, vehicle, and test reagents were added, as appropriate, but cells were omitted.

### Quantitative Real-Time RT-PCR

Quantitative real-time RT-PCR (qRT-PCR) was performed as described previously (Dong et al., 2010, 2015). Total RNA was isolated from cells incubated at different times as indicated, using RNeasy mini kits (Qiagen, Valencia, CA, USA) according to the manufacturer’s instructions. cDNA was synthesized using M-MLV reverse transcriptase (Promega, Madison, WI, USA). Primers were designed using ABI Primer Express Software v3.0. and synthesized by Integrated DNA Technologies, Inc. (Coralville, IA, USA) The primers used for the different mRNA expressions analyzed are presented in **Table 1**. qRT-PCR was performed using an ABI 7500 fast system and data analyzed using 7500 fast system SDS software v3.0.

**Table 1:** Sequences of primers used for quantitative real-time RT-PCR.

Name	Sequence
GR	Forward: AGCCATTGTCAAGAGCGAAC
	Reverse: TGATTGGTGATGATTTCAGCTA
GR1A3	Forward: GCCTGGCTCCTTTCCTCAA
	Reverse: CAGGAGTTAATGATTCTTTGGAGTCC
GR1B	Forward: GCCCAGATGATGCGGTG
	Reverse: TCTACCAGGAGTTAATGATTCTTTGGA
GR1C	Forward: GGGAACTGCGGACGGTG
	Reverse: GGAGTTAATGATTCTTTGGAGTCCA
Bim	Forward: ACAGAGCCACAAGACAGGAG
	Reverse: CCATTGCACTGAGATAGTGGTTG
Bad	Forward: CGGAGGATGAGTGACGAGTT
	Reverse: CCACCAGGACTGGAAGACTC
RPL19	Forward: GAGAAACGGCTGGATGATAGC
	Reverse: TGGTTAGGCTCTTGTACTACTGG

### Western Immunoblot Analysis

Western immunoblot analysis was performed as described previously (Dong et al., 2010, 2015; Vang et al., 2013). CEM and MM.1 cells were centrifuged at 300 × *g* for 5 min at the appropriate time points after treatments, washed twice with ice-cold PBS, and lysed in 100 μl RIPA buffer (50 mM Tri-HCl, pH 7.4, 150 mM NaCl, 1 mM EDTA, 1% NP-40, 0.25% Na-deoxycholate, and 1:100 protease inhibitor cocktail). Protein concentration was determined using a Micro BCA Protein Assay Kit (Pierce, Rockford, IL, USA). Equal amounts of protein were loaded and run on 12% SDS-PAGE gels. Proteins were then transferred onto Immobilon-p Transfer Membrane (Millipore). Membranes were blocked with 5% non-fat dry milk in Tris-buffered saline for 1 h at room temperature and probed with primary antibody overnight at 4°C, washed three times with TBS-T buffer, and incubated with horseradish peroxidase-conjugated secondary antibody at a final dilution of 1:5000 and then washed three more times. Proteins were visualized with SuperSignal West Femto maximum Sensitivity Substrate (Pierce, Rockford, IL, USA) and densities of the bands determined using either a UVP BioImaging system (St. Upland, CA, USA) or a Gene-snap Bioimaging system (Syngene, Frederick, MD, USA) with associated software. Blots were stripped and reprobed with GADPH antibody for normalization. Quantitative comparisons of protein expression following different treatments were calculated by determining the ratio of the target protein band density/GAPDH band density for each given treatment and time, divided by the ratio of the target protein band density/GAPDH band density for the untreated control for that given time.

## Results

### cAMP Signaling Induces Apoptosis of Both Glucocorticoid-Sensitive and Glucocorticoid-Resistant CEM and MM.1 Cell Lines

**FIGURE 1:**
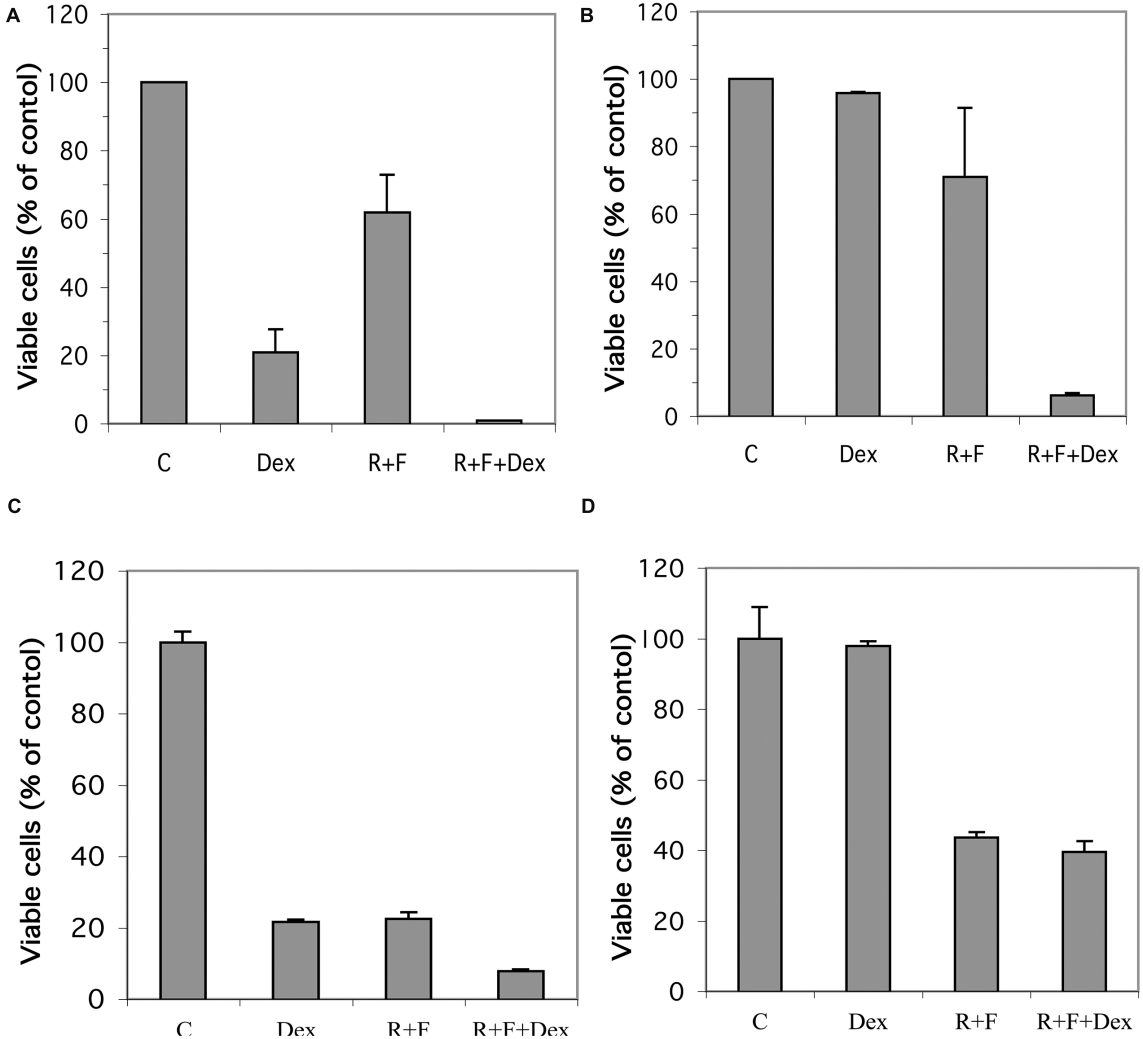
**Effect of dexamethasone, rolipram, and forskolin on viability of glucocorticoid-sensitive and glucocorticoid-resistant CEM and MM.1 cells. (A)** CEM-S2 cells, **(B)** CEM-R8 cells, **(C)** MM.1S cells, and **(D)** MM.1R cells were treated with 1 μM dexamethasone (Dex), 10 μM rolipram plus 10 μM forskolin (R+F), or 1 μM dexamethasone plus 10 μM rolipram plus 10 μM forskolin (R+F+Dex) for 72 h. Cell viability was then determined by the MTS method. Data represent the mean ± SD of at least two independent experiments assayed in triplicate.

Established glucocorticoid-sensitive and glucocorticoid-resistant CEM and MM.1 cell lines were maintained in culture and the effects of stimulation of the glucocorticoid and cAMP signaling pathways on viability of these cells was examined. As shown in **Figure 1A**, stimulation of the glucocorticoid signaling pathway by dexamethasone (1 μM) decreased the viability of the glucocorticoid-sensitive CEM-S2 cells by 78% after 72 h, but had no effect on the viability of the glucocorticoid-resistant CEM-R8 cells (**Figure 1B**). In contrast, stimulation of the cAMP signaling pathway by the adenylyl cyclase activator forskolin (10 μM) plus the PDE4 inhibitor rolipram (10 μM) decreased the viability of both CEM-S2 and CEM-R8 cells (40 and 30% inhibition, respectively, **Figures 1A,B**). Additionally, these effects of forskolin and rolipram on cell viability were greatly potentiated by dexamethasone, resulting in 98 and 95% decrease in cell viability of CEM-S2 and CEM-R8 cells respectively when both the cAMP and glucocorticoid signaling pathways were stimulated concurrently (**Figures 1A,B**). Very similar to results seen with CEM leukemic cells, stimulation of the glucocorticoid signaling pathway by dexamethasone (1 μM) also decreased the viability of the glucocorticoid-sensitive MM.1S multiple myeloma cells by 78% after 72 h (**Figure 1C**), and had no effect on the viability of the glucocorticoid-resistant MM.1R cells (**Figure 1D**). Additionally, also similar to CEM cells, stimulation of the cAMP signaling pathway by forskolin (10 μM) and rolipram (10 μM) greatly decreased the viability of both MM.1S cells (78% inhibition) and MM.1R cells (56% inhibition). The effects of forskolin and rolipram on cell viability were potentiated by the addition of dexamethasone in MM.1S cells, where inhibition of cell viability was increased from 78 to 92% (**Figure 1C**), although, unlike glucocorticoid-resistant CEM-R8 cells, there was only a small potentiation in glucocorticoid-resistant MM.1R cells where inhibition of cell viability was increased from 56 to 60% (**Figure 1D**). Analysis of the cells following these treatments by direct microscopic visualization revealed cell loss and cell shrinking, and by gel analysis of isolated genomic DNA revealed DNA ladder patterns, indicating that loss of cell viability is consistent with apoptosis of the cells (data not shown). These results indicate that the glucocorticoid-resistant CEM-R8 leukemic and MM.1R multiple myeloma cell lines, while completely resistant to the effects of glucocorticoids, are still able to be killed by stimulation of the cAMP signaling pathway. Further, stimulation of the cAMP and glucocorticoid signaling pathways together act synergistically to decrease viability of both glucocorticoid-sensitive and resistant CEM cells and glucocorticoid-sensitive MM.1 cells.

### Forskolin Acts through Stimulation of the cAMP Signaling Pathway

**FIGURE 2:**
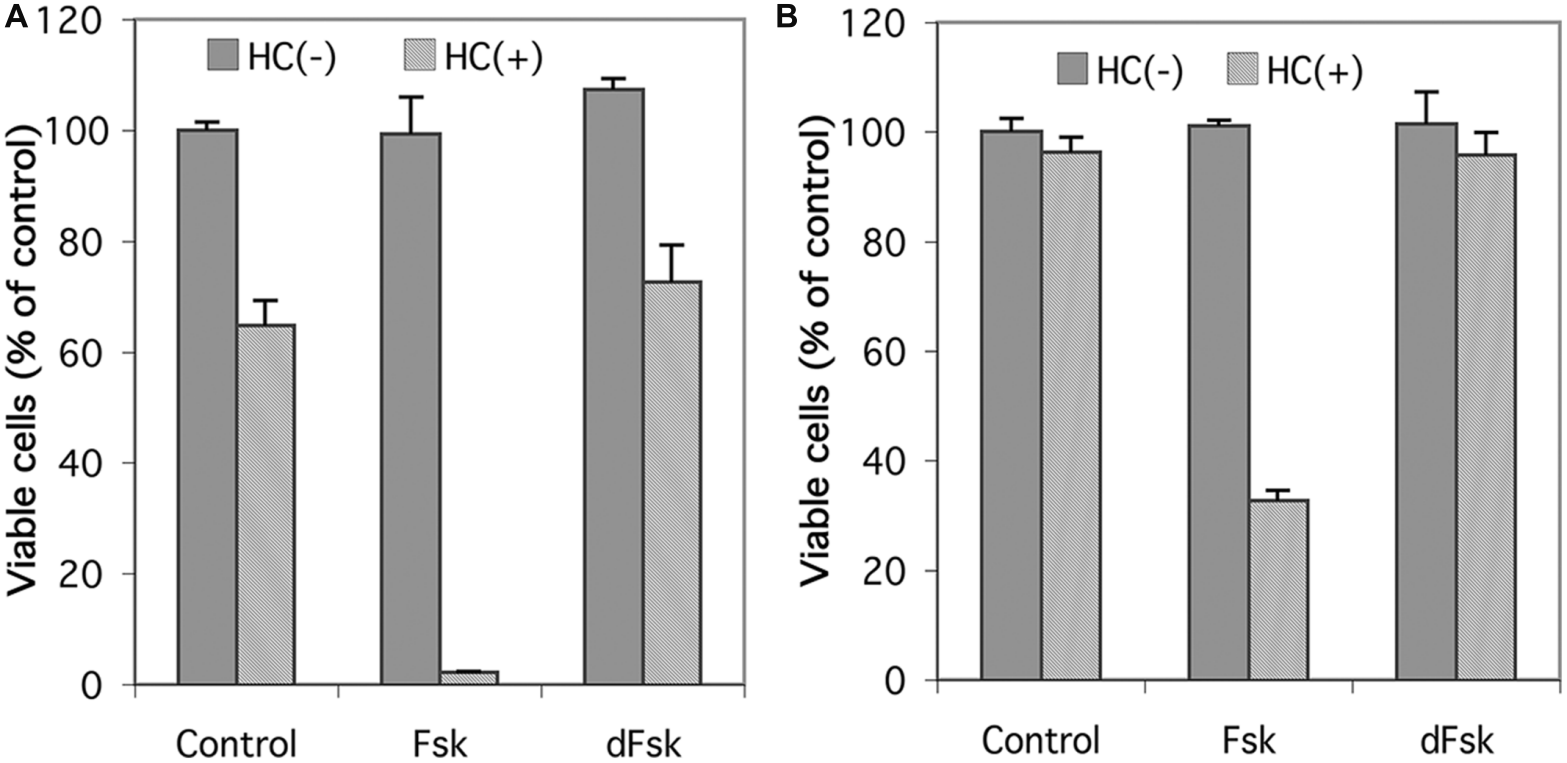
**Effect of 1,9-dideoxyforskolin on CEM cells.** Cell viability of CEM-S2 cells **(A)** and CEM-R8 cells **(B)**. Cells were treated with 10 μM forskolin (Fsk) or 10 μM 1,9-dideoxyforskolin (dFsk) in the presence or absence of 10 μM hydrocortisone (HC) for 72 h. After treatment, cell viability was determined by MTS assay. Data represent the mean ± SD of at least two independent experiments assayed in triplicate.

Inasmuch as forskolin has been reported to produce effects independent of its activation of adenylyl cyclase (Ding and Staudinger, 2005; Riddell et al., 2013; Angel-Chavez et al., 2015), we compared forskolin with 1,9-dideoxyforskolin, an analog of forskolin that does not stimulate adenylyl cyclase, but maintains many of the other pleiotropic effects of forskolin, for their ability to synergize with glucocorticoids to induce cell death in CEM cells. As seen in **Figure 2**, neither forskolin nor 1,9-dideoxyforskolin by themselves have any effect on viability of CEM-S2 or CEM-R8 cells; however, forskolin synergizes with hydrocortisone to induce cell death in CEM-S2 and CEM-R8 cells, whereas 1,9-dideoxyforskolin does not. This indicates that forskolin is most likely acting through stimulation of the cAMP signaling pathway, and not through other pleiotropic effects.

### Expression of the Glucocorticoid Receptor Alpha (GR) Gene Product is Little Changed by Stimulation of the cAMP and Glucocorticoid Signaling Pathways in CEM cells and Greatly Downregulated in MM.1 Cells

**FIGURE 3:**
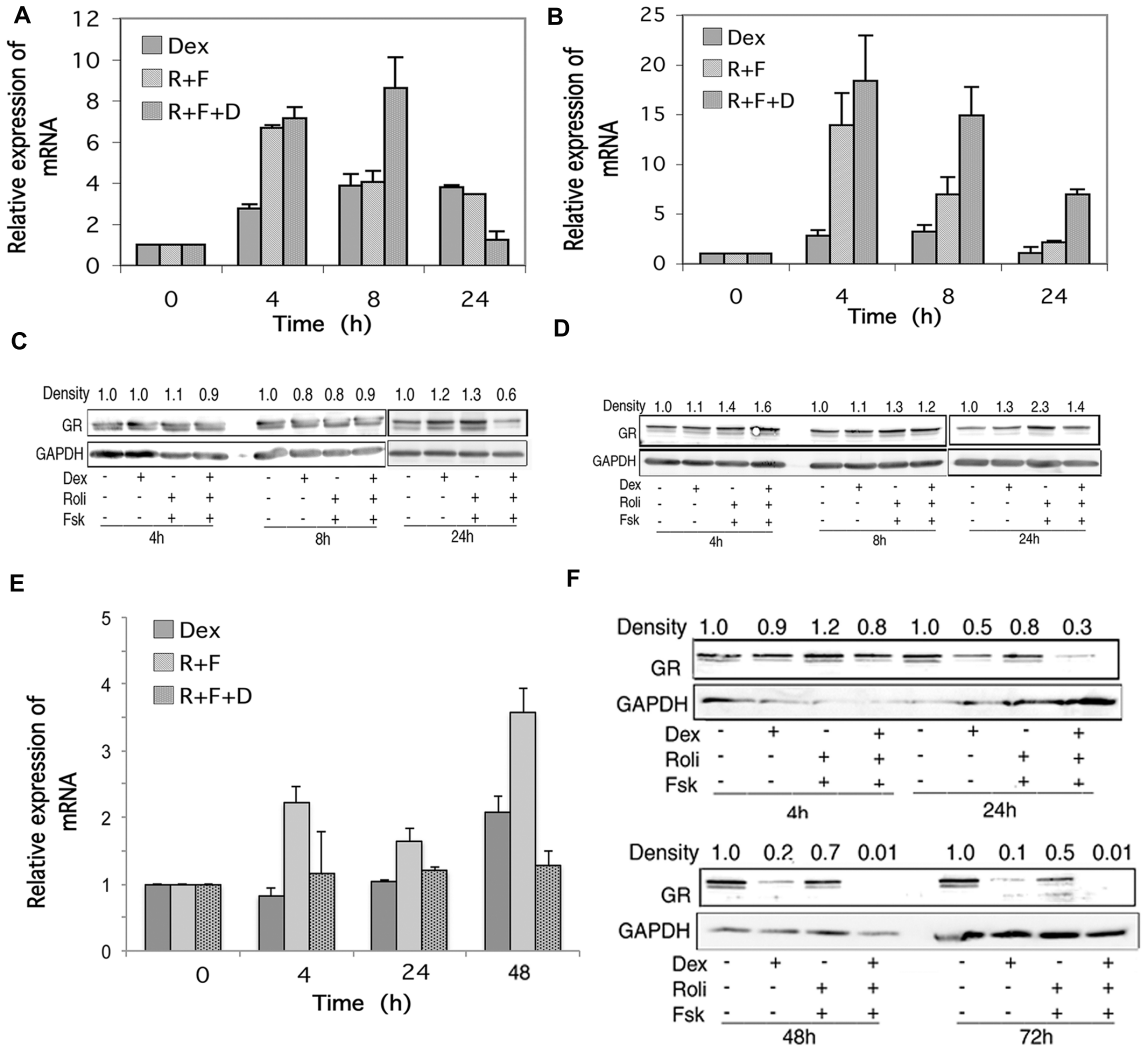
**Glucocorticoid receptor (GR) expression in CEM and MM.1 cells. (A,B,E)** Quantitative real-time PCR analysis of GR mRNA expression in CEM-S2 **(A)**, CEM-R8 **(B)**, and MM.1S **(E)** cells, following treatment with 1 μM dexamethasone (Dex), 10 μM rolipram plus 10 μM forskolin (R+F), or 1 μM dexamethasone plus 10 μM rolipram plus 10 μM forskolin (R+F+D) at different times as indicated. Data represent the mean ± SD of at least two independent experiments assayed in triplicate, shown as fold change relative to control. **(C,D,F)** Western blot analysis of GR protein expression in CEM-S2 **(C)**, CEM-R8 **(D)**, and MM.1S **(F)** cells following treatment at different times with dexamethasone and/or rolipram and forskolin as indicated. Equal amounts of protein (20 μg) from whole cell lysates were added per lane. Data shown represents one of at least two independent experiments with similar results. The numbers at the top of the Western blot images represent the GR band densities following different treatments relative to control, normalized based on the density of the GAPDH housekeeping protein, calculated as described in Section “Materials and Methods.”

RNA transcript and protein expression for the alpha form of the GR was examined by qRT-PCR and Western blot analysis in response to stimulation of the cAMP and glucocorticoid signaling pathways. As shown in **Figures 3A,B**, stimulation of the cAMP signaling pathway by forskolin and rolipram and the glucocorticoid pathway by dexamethasone induced transcription of the mRNA for GR in CEM cells. When protein product for GR was analyzed by Western blot, however, little change was seen in its expression following stimulation of the cAMP signaling pathway, except for some (1.3–2.3-fold) induction at the late, 24 h time point, for both glucocorticoid-sensitive and resistant CEM cells, and this induction of GR was attenuated by simultaneous stimulation of the glucocorticoid pathway with dexamethasone (**Figures 3C,D**). The doublets seen in the Western blot immunostaining for GR alpha (**Figures 3C,D,F**) of Mr 94 kDa and Mr 91 kDa, represent the two forms of GR alpha, termed GR-A and GR-B, produced by alternative translation of the GR alpha gene, with the 91 kDa form produced from an internal ATG codon corresponding to met27 (Yudt and Cidlowski, 2001). In MM.1S cells, cAMP and glucocorticoid signaling produced either no change or small increases in mRNA for GR (**Figure 3E**), but GR protein was greatly diminished in response to dexamethasone treatment, and further diminished to the point of being nearly absent at the 48 and 72 h time points, when both the glucocorticoid and cAMP signaling pathways were stimulated together (**Figure 3F**). The MM.1R cell line has been reported to mainly express truncated forms of the GR with non-functional hormone binding domains, and as such, produces very little full length GR product (Moalli et al., 1992). Consistent with this, we were unable to detect any expression for GR in MM.1R cells under any conditions (data not shown).

### Stimulation of the cAMP Signaling Pathway Induces the 1A, 1B, and 1C Promoters for GR in CEM and MM.1 Cells

**FIGURE 4:**
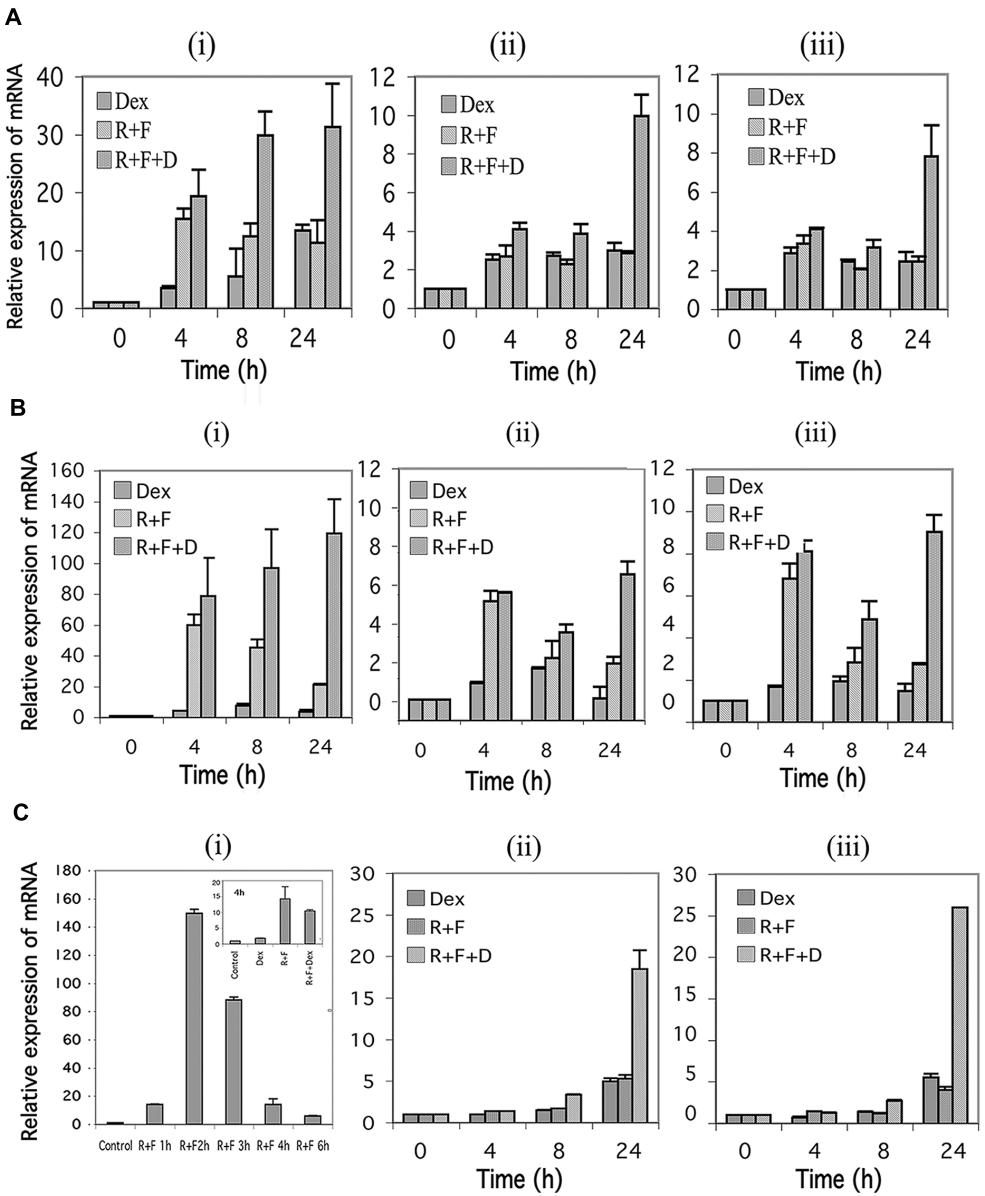
**Glucocorticoid receptor alpha (GR) promoter 1A, 1B, and 1C expression in CEM and MM.1 cells.** Quantitative real-time PCR analysis of GR1A3, 1B and 1C mRNA expression in both CEM and MM.1 cells. **(A)**, GR1A3(i), 1B(ii), and 1C(iii) expression in CEM-S2 cells. **(B)**, GR1A3(i), 1B(ii), and 1C(iii) expression in CEM-R8 cells. **(C)**, GR1A3(i), 1B(ii), and 1C(iii) expression in MM.1S cells. Points were taken following treatment with 1 μM dexamethasone (Dex), 10 μM rolipram plus 10 μM forskolin (R+F), or 1 μM dexamethasone plus 10 μM rolipram plus 10 μM forskolin (R+F+D) for different times as indicated. Data represent the mean ± SD of at least two independent experiments assayed in triplicate.

In humans, the GR is transcribed from at least three different promoters, termed 1A, 1B, and 1C, with 1A further exhibiting three different splice sites designated 1A1, 1A2, and 1A3 (Yudt and Cidlowski, 2002). Using primers specific for the 1A3, 1B, and 1C promoter regions, we examined the effect of stimulation of the cAMP signaling pathway on expression of the three GR promoters. As seen in **Figures 4Ai,Bi**, expression of the 1A3 promoters for CEM-S2 and CEM-R8 cells were highly induced by stimulation of cAMP signaling, and this was potentiated by glucocorticoid signaling to yield inductions of the 1A3 promoter of as much as ≈30–120-fold by 24 h. The 1B and 1C promoters in CEM cells were also induced 6–8-fold at 24 h by combined cAMP and glucocorticoid signaling (**Figures 4Aii,iii,4Bii,iii**). The 1A3 promoter was transiently induced by forskolin and rolipram in MM.1S cells. A 150-fold induction of the 1A3 promoter was seen in MM.1S cells at 2 h after forskolin and rolipram treatment, after which the enhanced expression diminished to only sixfold by 6 h (**Figure 4Ci**), and expression of 1A3 was then undetectable at 24 and 48 h (not shown). Dexamethasone by itself had little effect on the expression of the 1A3 promoter in MM.1S cells, but, as with protein expression of the GR itself, dexamethasone attenuated the induction of the 1A3 promoter by forskolin and rolipram (**Figure 4Ci** insert). The effect of cAMP signaling on expression of the 1B and 1C promoters in MM.1S cells was quite different from that of the 1A3 promoter. Forskolin and rolipram had no effect on expression of the 1B and 1C promoters until 48 h, at which time they induced expression of both of these promoters by 4–5-fold. Dexamethasone by itself also had no effect on expression of the 1B and 1C promoters until 48 h, at which time it induced expression of both of these promoters also by about fivefold. However, whereas dexamethasone attenuated the induction of the 1A3 promoter by forskolin and rolipram, in contrast, it greatly potentiated the induction of the 1B and 1C promoters, resulting in 19–26-fold induction of both of these promoters (**Figure 4Cii,iii**).

### Bim Expression is Induced by Stimulation of the cAMP and Glucocorticoid Signaling Pathways in Glucocorticoid-Sensitive and Resistant CEM Cells and in Glucocorticoid-Sensitive MM.1 Cells, but not in Glucocorticoid-Resistant MM.1 Cells

**FIGURE 5:**
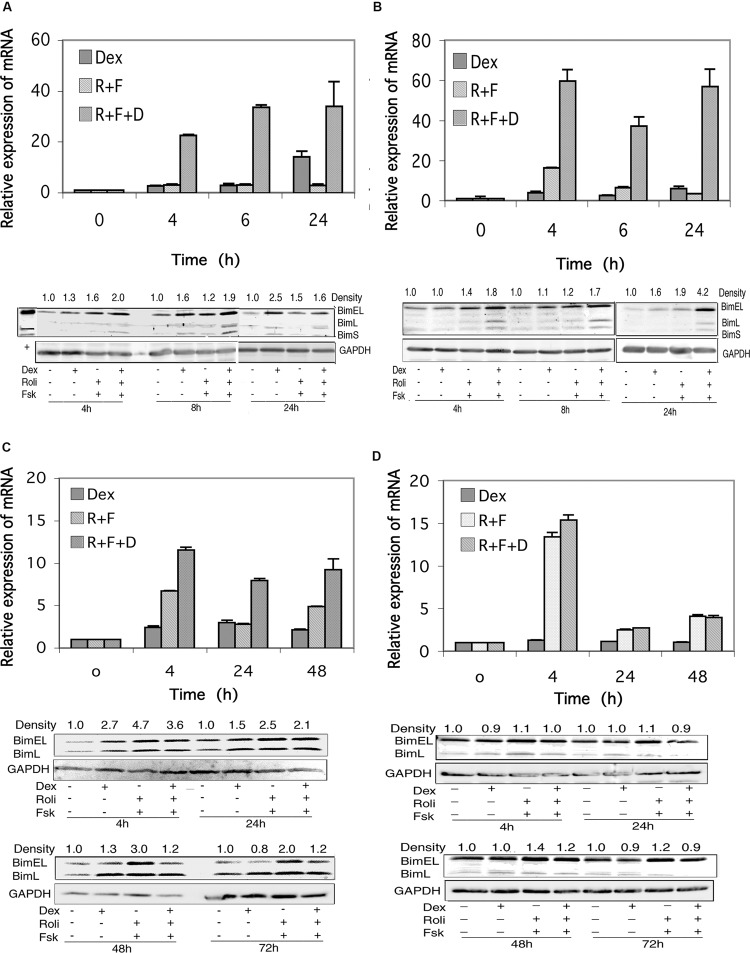
**Bim expression in CEM and MM.1 cells. (A)** Quantitative real-time PCR analysis of Bim mRNA expression in CEM-S2 cells (top), at different times following treatment with 1 μM dexamethasone (Dex), 10 μM rolipram plus 10 μM forskolin (R+F) or 1 μM dexamethasone plus 10 μM rolipram plus 10 μM forskolin (R+F+D) as indicated. Data represent the mean ± SD of at least two independent experiments assayed in triplicate, shown as fold change relative to control. (bottom), Western blot analysis of expression of Bim protein. Cells were treated at different times with dexamethasone and/or rolipram and forskolin as indicated, and Bim protein expression was determined by immunoblots of whole cell lysates. Equal amounts of protein (20 μg) were loaded per lane. Data shown represents one of at least two independent experiments with similar results. The numbers at the top of the Western blot images represent the BimEL band densities following different treatments relative to control, normalized based on the density of the GAPDH housekeeping protein, calculated as described in Section “Materials and Methods.” **(B)** CEM-R8 cell Bim mRNA (top) and protein (bottom) expression. **(C)** Bim mRNA (top) and protein (bottom) expression in MM.1S cells. **(D)** Bim mRNA (top) and protein (bottom) expression in MM.1R cells. Conditions were as in **(A)**.

RNA transcript and protein expression for the BH3-only proapoptotic protein, Bim, was examined by qRT-PCR and Western blot analysis in response to stimulation of the cAMP and glucocorticoid signaling pathways. As shown in **Figures 5A,B**, mRNA expression for Bim is induced several fold by cAMP and glucocorticoid signaling in both CEM-S2 and CEM-R8 cells, at all time points examined, and this induction is greatly potentiated, yielding inductions of ≈20–60-fold, when both signaling pathways are stimulated together.

Expression of Bim protein in response to stimulation of these signaling pathways reflected the changes seen in Bim mRNA levels, for the most part. Bim can be expressed as three different splice variants, an extra long form of Mr ≈24 kDa (BimEL), a long form of Mr ≈21 kDa (BimL), and a short form of Mr ≈19 kDa (BimS), and CEM cells express all three of these Bim protein products. As seen in **Figures 5A,B**, all three Bim protein products are induced to some extent following stimulation of cAMP or glucocorticoid signaling, and the induction is more pronounced (1.6–4.2-fold stimulation of BimEL) when both signaling pathways are stimulated together.

As shown in **Figure 5C**, stimulation of the cAMP signaling pathway with forskolin and rolipram induced expression of the mRNA for Bim in MM.1S cells by sevenfold at 4 h, threefold at 24 h, and fivefold at 48 h. Stimulation of the glucocorticoid pathway by dexamethasone also induced expression of Bim mRNA in MM.1S cells by 2–3-fold at each of these time points. When both signaling pathways were activated by adding forskolin, rolipram, and dexamethasone together, induction of Bim mRNA was potentiated, resulting in induction of 12-fold at 4 h, eightfold at 24 h, and ninefold at 48 h (**Figure 5C**). Analysis of the effects of these agents on Bim mRNA transcript in MM.1R cells showed that stimulation of the cAMP signaling pathway with forskolin and rolipram also induced Bim mRNA expression in these cells, by 13-fold at 4 h, twofold at 24 h, and fourfold at 48 h (**Figure 5D**). However, in contrast to MM.1S cells, and in contrast to the effects of cAMP signaling, stimulation of the glucocorticoid signaling pathway with dexamethasone in MM.1R cells had no effect at all on the expression of Bim mRNA, and little or no effect on the induction seen in response to forskolin and rolipram (**Figure 5D**).

In contrast to CEM (**Figures 5A,B**) and Hut78 (data not shown) T leukemic cell lines, which visibly express all three forms of Bim protein, expression of BimS in MM.1 cells was far less than that of BimEL and BimL and was only detectable if the Western blots were greatly overexposed (data not shown). In MM.1S cells, expression of the BimEL and BimL splice variants were induced at most of the time points measured, 4, 24, 48, and 72 h, in response to dexamethasone, forskolin plus rolipram, or all three agents added together (**Figure 5C**). However, induction of Bim protein in MM.1S cells was somewhat higher following stimulation of the cAMP signaling pathway alone (2–4.7-fold) than following concurrent stimulation of the cAMP and glucocorticoid signaling pathways (1.2–3.6-fold), and at all time points examined stimulation of the glucocorticoid signaling pathway actually attenuated the induction effect of cAMP on Bim protein expression. In contrast to MM.1S cells, in MM.1R cells, there was little change in the expression of BimEL and BimL following stimulation of the cAMP and glucocorticoid signaling pathways, either alone or together (**Figure 5D**).

### Bad Expression is Little Changed in CEM Cells in Response to Stimulation of the cAMP and Glucocorticoid Signaling Pathways, but is Highly Induced in MM.1 Cells

**FIGURE 6:**
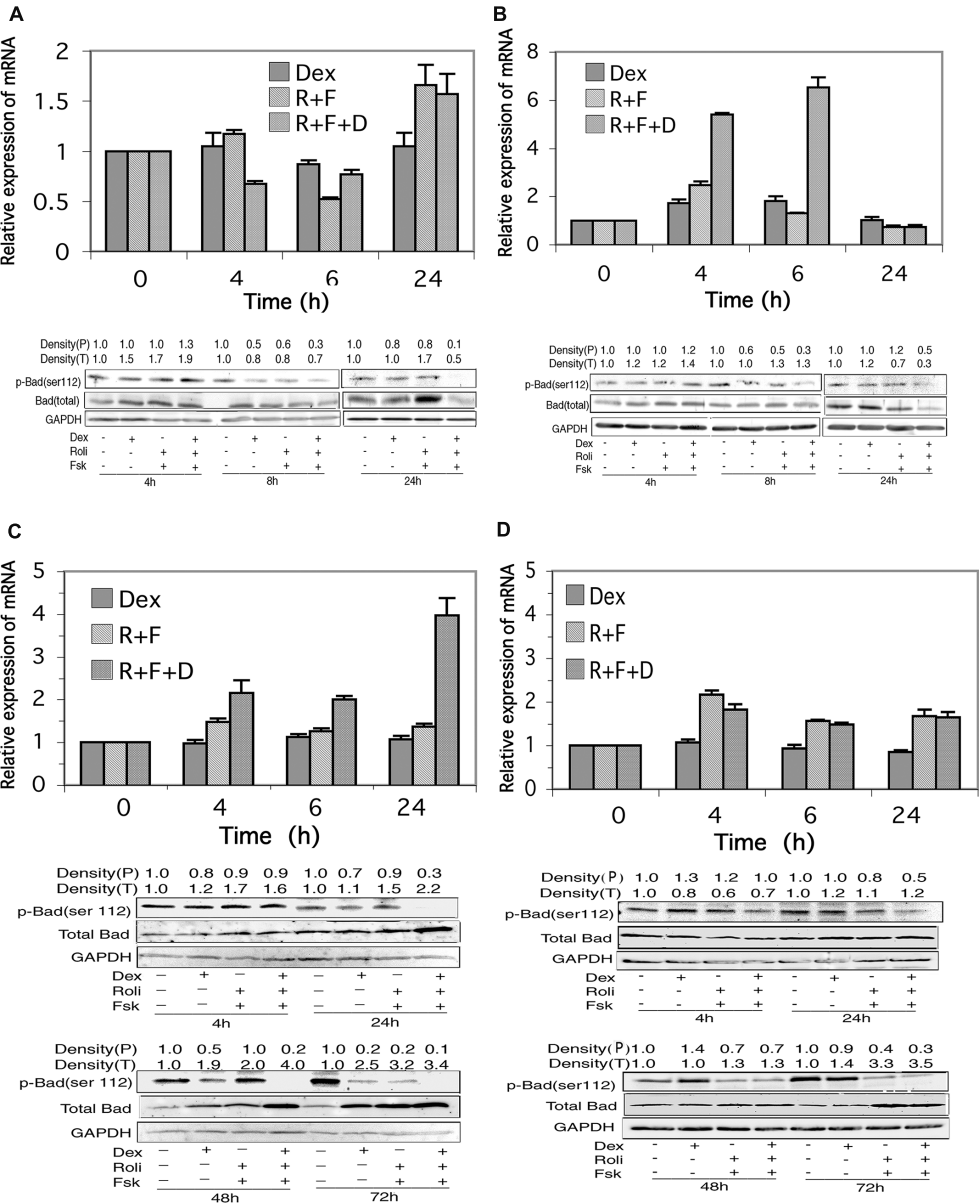
**Bad expression in CEM and MM.1 cells.** Top panels: quantitative real-time PCR analysis of Bad mRNA at different times after treatment with 1 μM dexamethasone (Dex), 10 μM rolipram plus 10 μM forskolin (R+F), or 1 μM dexamethasone plus 10 μM rolipram plus 10 μM forskolin (R+F+D) as indicated. Data represent the mean ± SD of at least two independent experiments assayed in triplicate, shown as fold change relative to control. Bottom panels: Western blot analysis of expression of total Bad and p-Bad (ser112) protein. Cells were treated at different times with dexamethasone and/or rolipram and forskolin as indicated, and Bad protein expression was determined by immunoblots of whole cell lysates. An equal amount of protein (20 μg) was loaded per lane. Densities of phosphorylated Bad (P) and total Bad (T) are given at the top of the Western blot panels, calculated as described in Section “Materials and Methods.” Data shown represents one of at least two independent experiments with similar results. **(A)** CEM-S2 cells; **(B)** CEM-R8 cells; **(C)** MM.1S cells, and **(D)** MM.1R cells.

RNA transcript and protein expression for the BH3-only proapoptotic protein, Bad, was examined by qRT-PCR and Western blot analysis in response to stimulation of the cAMP and glucocorticoid signaling pathways. As shown in **Figure 6A**, in CEM-S2 cells, stimulation of the glucocorticoid signaling pathway with dexamethasone produced no effect on Bad mRNA expression at any time point examined, and stimulation of the cAMP signaling pathway with forskolin and rolipram produced a small increase in Bad mRNA expression, about 1.5-fold, only at the 24 h time point, with or without dexamethasone. Similarly, as shown in **Figure 6B**, in CEM-R8 cells, stimulation of either the cAMP or glucocorticoid signaling pathways produced little change in Bad mRNA expression, although combined stimulation of the glucocorticoid and cAMP signaling pathways produced a 5–6-fold increase in the expression of Bad mRNA at the 4 and 6 h time points. Also as shown in **Figures 6A,B**, expression of total Bad protein in CEM cells was also little affected by these signaling pathways. Stimulation of the glucocorticoid and cAMP signaling pathways produced only very small increases in the expression of total Bad protein, seen mostly at the 4 h time point, where in CEM-S2 cells, Bad protein increased 1.5-fold in response to dexamethasone, 1.7-fold in response to forskolin plus rolipram, and 1.9-fold when all three agents were added together. In CEM-R8 cells at 4 h Bad protein increased 1.2-fold in response to dexamethasone, 1.2-fold in response to forskolin plus rolipram, and 1.4-fold when all three agents were added together.

In MM.1S and MM.1R cells, as shown in **Figures 6C,D**, stimulation of the glucocorticoid signaling pathway alone by dexamethasone had no effect on expression of the mRNA for Bad at any of the time points measured. Stimulation of the cAMP pathway with forskolin and rolipram resulted in a small upregulation of Bad mRNA, ranging from 1.3 to 1.5-fold in MM.1S cells and 1.6–2.2-fold in MM.1R cells at the different time points measured. Addition of dexamethasone to forskolin and rolipram produced no further change in Bad mRNA expression in MM.1R cells, but potentiated the effects of cAMP signaling in MM.1S cells, such that the expression levels of Bad mRNA in MM.1S cells were increased to 2.2-fold at 4 h, twofold at 24 h, and fourfold at 48 h (**Figures 6C,D**).

Western blot analysis of total Bad protein revealed appreciable increases in expression in MM.1S cells in response to stimulation of cAMP and glucocorticoid signaling. Bad protein expression increased 1.1–1.7-fold by independent stimulation of the cAMP or glucocorticoid signaling pathways at 4 h and 24 h, and increased further to 2.2-fold at 24 h when both signaling pathways were stimulated by addition of dexamethasone and forskolin plus rolipram together (**Figure 6C**). At 48 h, expression of Bad protein was increased 1.9-fold by dexamethasone and twofold by forskolin plus rolipram treatment, and its expression was increased further to fourfold when all three agents were added together to stimulate both signaling pathways (**Figure 6C**). At 72 h, Bad expression was increased 2.5-fold by dexamethasone, 3.2-fold by forskolin plus rolipram, and 3.4-fold when all three agents were added together (**Figure 6C**). Similarly, in MM.1R cells, Bad expression was also induced by stimulation of these signaling pathways, with the largest effects seen at 72 h, where Bad expression was increased 1.4-fold by dexamethasone, 3.3-fold by forskolin plus rolipram, and 3.5-fold by all three agents added together (**Figure 6D**). Hence, although total Bad protein is little changed in CEM-S2 and CEM-R8 cells in response to stimulation of cAMP and glucocorticoid signaling, it is considerably induced in MM.1 cells, both in the MM.1S sensitive and MM.1R resistant cells, both by stimulation of the cAMP and glucocorticoid signaling pathways independently, and to an even greater extent when both signaling pathways are stimulated together.

### Stimulation of the cAMP Signaling Pathway Alters the Phosphorylation State of Bad in Glucocorticoid-Sensitive and Resistant CEM and MM.1 Cells

The activity of Bad, as a proapoptotic protein, is regulated by its state of phosphorylation. Phosphorylation of Bad on several sites, especially Ser112, promotes its association with 14-3-3 protein and sequesters it in an inactive state. Dephosphorylation of Bad at these sites promotes its dissociation from 14-3-3 allowing it to interact with mitochondria and promote apoptosis (Chiang et al., 2001; Moon and Lerner, 2003). We therefore investigated the state of phosphorylation of Bad following stimulation of the glucocorticoid and cAMP signaling pathways. As shown in **Figures 6A,B**, although there was little change in total Bad protein in CEM cells following stimulation of the cAMP and glucocorticoid pathways, stimulation of these two signaling pathways together resulted in dephosphorylation of Bad at ser112 in both the glucocorticoid-sensitive and resistant CEM cell lines. Additionally, very similar to the effect seen with CEM cells, stimulation of the cAMP and glucocorticoid signaling pathways also led to a dramatic dephosphorylation of Bad on ser112 in both the MM.1S and MM.1R cells (**Figures 6C,D**). Hence, one common result of the stimulation of the cAMP and glucocorticoid pathways in both the glucocorticoid-sensitive and resistant CEM and glucocorticoid-sensitive and resistant MM.1 cells is the activation of Bad via its dephosphorylation on ser112.

### Model for the Synergy between cAMP and Glucocorticoid Signaling Pathways on Induction of Apoptosis in Leukemic and Multiple Myeloma Cells

**FIGURE 7:**
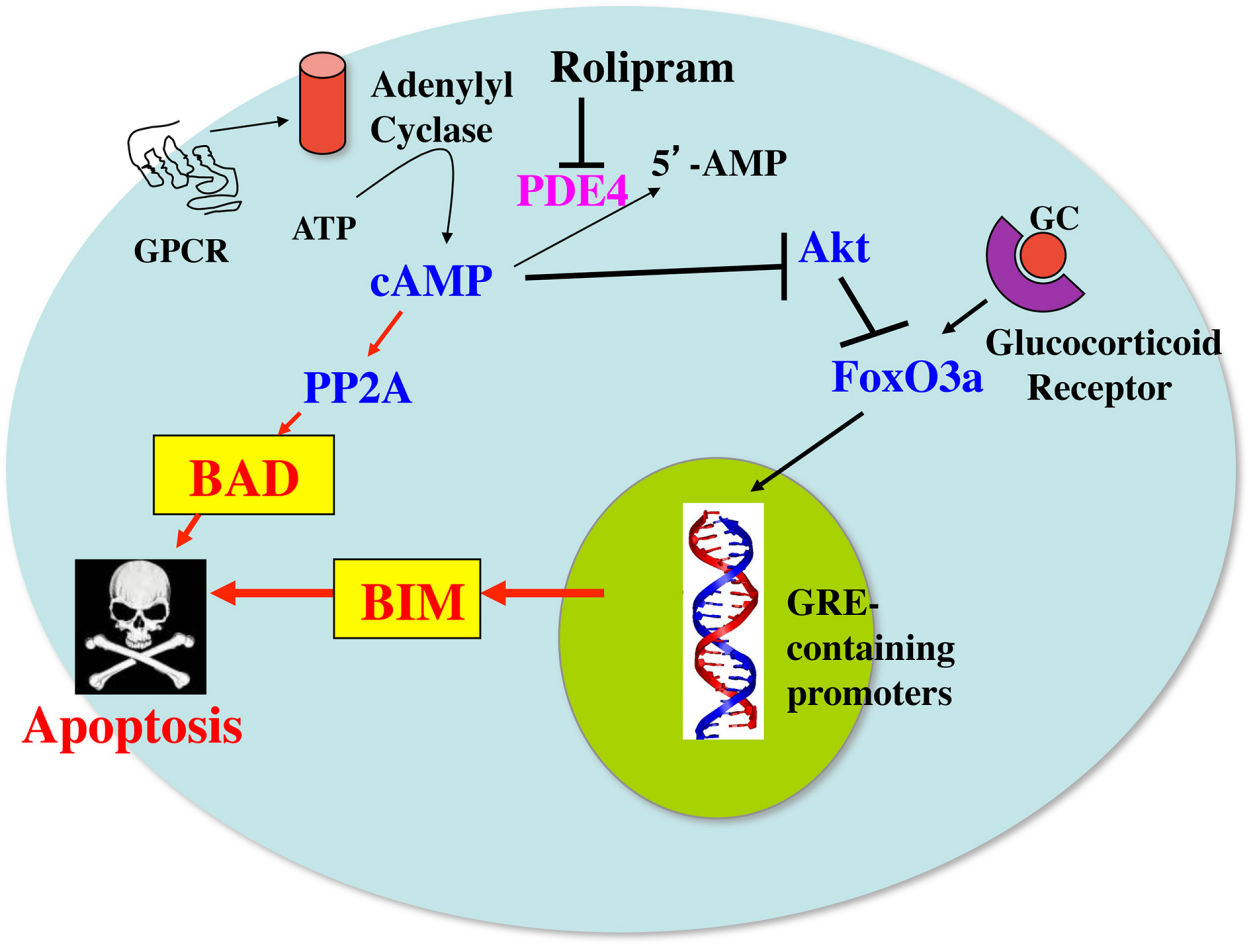
**Model for the synergy between cAMP and glucocorticoid signaling pathways on induction of apoptosis in leukemic and multiple myeloma cells.** GC, glucocorticoids; GPCR, G protein coupled receptor; PP2A, protein phosphatase 2A; PDE4, phosphodiesterase4; GRE, glucocorticoid-responsive element.

A model for how cAMP and glucocorticoid signaling might synergize with each other to induce apoptosis and overcome glucocorticoid resistance in these cells is presented in **Figure 7**. In this model it is proposed that glucocorticoids upregulate the expression of Bim through a Fox03a-dependent mechanism. Phosphodiesterase inhibitors, such as the PDE4 inhibitor rolipram, or agents that activate adenylyl cyclase through G protein coupled receptors, stimulate cAMP signaling which can lead to activation of the protein phosphatase 2A. The activated protein phosphatase 2A then dephosphorylates Bad on ser112, resulting in its dissociation from 14-3-3 chaperone protein and its translocation to the mitochondria, where it acts as a sensitizer by binding to antiapoptotic proteins such as Bcl-2 in leukemic cells and Mcl-1 in multiple myeloma cells, and releasing Bim from its association with these proteins. The released Bim then binds to Bax and the complex acts to permeabilize the mitochondrial membrane, leading to apoptosis. In some cells, as shown in this study for the MM.1S and MM.1R multiple myeloma cells, the increased cAMP signaling not only activates Bad, but also upregulates its expression. Further, the increased cAMP can also inhibit the growth promoting kinase, Akt, which disinhibits Fox03a, leading to further upregulation of Bim. The increased Bim, as an activator protein, primes the cells for death in response to the translocation of the dephosphorylated, activated Bad sensitizer protein to the mitochondria, leading to a synergistic induction of apoptosis and overcoming glucocorticoid resistance.

## Discussion

Resistance to glucocorticoids is a major problem in the treatment of leukemia and multiple myeloma (Haarman et al., 2003; Frankfurt and Rosen, 2004; Schmidt et al., 2004; Ploner et al., 2005; Sionov et al., 2008; Bhadri et al., 2012). In this study we investigated changes in GR, and the BH3-only proapoptotic proteins, Bim and Bad, in cell line models of glucocorticoid-sensitive and resistant leukemia and multiple myeloma. Consistent with other reports (Krett et al., 1997; Medh et al., 1998; Ogawa et al., 2002; Tiwari et al., 2005; Lerner and Epstein, 2006; Dong et al., 2010; Follin-Arbelet et al., 2011) in the cell models we examined here, we found that both the glucocorticoid-sensitive and resistant leukemia and multiple myeloma cell lines could be killed by stimulation of the cAMP signaling pathway, and that stimulation of the cAMP signaling pathway acted synergistically with glucocorticoids to induce cell death of both the glucocorticoid-sensitive and resistant CEM leukemic cell lines and the glucocorticoid-sensitive MM.1S multiple myeloma cell line. A synergistic effect of cAMP and glucocorticoid signaling was not seen with the MM.1R, glucocorticoid-resistant cell line, most likely as a result of the MM.1R cell line expressing a truncated form of GR (Moalli et al., 1992).

Although loss or mutations in GR occur in a number of glucocorticoid-resistant cell lines, this only rarely occurs in primary cells from most patients, and thus appears not to account for the prevalence of glucocorticoid-resistance seen in patients treated with glucocorticoids (Bachmann et al., 2005, 2007; Tissing et al., 2006; Beesley et al., 2009). We examined changes in the expression of GR mRNA, GR promoters, and GR protein in glucocorticoid-sensitive and resistant leukemia and multiple myeloma cells in response to stimulation of cAMP and glucocorticoid signaling in these cells, and examined the correlation of these changes with induction of cell death in these cells following stimulation of these signaling pathways. Most cell types demonstrate down regulation of GR following glucocorticoid exposure, presumably as a feedback mechanism, although GR in some lymphoid leukemia cell lines has been reported to be upregulated (Tissing et al., 2006). It has also been observed in some cell lines that dexamethasone can dramatically alter GR protein levels without changing GR mRNA expression, leading to the suggestion that glucocorticoids may influence GR protein levels by other means besides transcriptional regulation, such as mRNA and GR protein stability (Okret et al., 1991). Effects of cAMP signaling on GR expression are also complex and can lead either to upregulation (Oikarinen et al., 1984; Dong et al., 1988) or down regulation (Sheppard et al., 1991) depending on the cell type. A study from the Lerner laboratory showed that stimulation of the cAMP signaling pathway with PDE4 inhibitors upregulated GR transcript levels in B-CLL cells but not T-CLL cells, Sezary cells or normal circulating T or B cells, monocytes, or neutrophils (Meyers et al., 2007). In this same study, it was shown that glucocorticoids reduce GR mRNA levels in B-CLL and that co-treatment with PDE4 inhibitors maintains GR mRNA levels above baseline. In our current study, although we see some increased expression of GR mRNA following stimulation of cAMP and glucocorticoid signaling, we see almost no change, only very small modest increases in GR protein at later time points, in both glucocorticoid-sensitive CEM-S2 and glucocorticoid-resistant CEM-R8 cells following stimulation of either cAMP signaling, stimulation of glucocorticoid signaling, or stimulation of both signaling pathways together. In contrast, in the MM.1S multiple myeloma cells, which are of B cell origin, stimulation of glucocorticoid signaling with dexamethasone greatly down regulated GR protein. However, unlike that seen in the B-CLL cells, co-stimulation of the cAMP signaling pathway enhanced this down regulation, rather than preventing it, leading to a profound reduction in the expression of GR.

Results from this study indicate that the cell death induced by cAMP signaling in these cells and the potentiation of glucocorticoid-induced cell death by cAMP signaling does not correlate with increased expression of GR protein, suggesting that the actions of these signaling pathways on viability of these cells is not mediated by changes in expression of GR. However, it is possible that alterations in GR may still play a role in the actions of these signaling pathways at the post-translational level, since GR has been shown to be a substrate for PKA (Haske et al., 1994), and thus its actions could be modified by cAMP signaling. Further, there is some evidence to suggest that translocation of GR from the cytosol to mitochondria may also play a role in mediating cell death (Sionov et al., 2006), and it is possible that this translocation could be influenced by these signaling pathways. Additionally, we found that cAMP signaling induced expression of the GR promoters in glucocorticoid-sensitive and resistant CEM cells and glucocorticoid-sensitive MM.1 cells, with induction of the 1A3 promoter stimulated more than 1B and 1C, and with further potentiation of this effect by dexamethasone in CEM cells. cAMP signaling was also shown to increase levels of the 1A3 promoter to a greater extent than the 1B promoter in B-CLL cells as well (Meyers et al., 2007). Inasmuch as expression of GR from the 1A promoter correlates best with T lymphocyte sensitivity to glucocorticoid-induced cell death (Purton et al., 2004), a shift to expression of more of the GR from the 1A3 promoter, relative to its expression from the other promoters, might also contribute to the cAMP sensitivity of these cells.

A number of studies have pointed to Bim as being an essential mediator of glucocorticoid-induced apoptosis of leukemic, lymphoma and myeloma cells, in that Bim is upregulated in these cells following glucocorticoid treatment, and down regulation of the expression of Bim inhibits glucocorticoid-induced apoptosis (Wang et al., 2003; Abrams et al., 2004; Bachmann et al., 2005; Ploner et al., 2005; Lu et al., 2006; Iglesias-Serret et al., 2007; Lopez-Royuela et al., 2010; Jiang et al., 2011; Zhao et al., 2011; Heidari et al., 2012; Jing et al., 2015). Additionally, stimulation of the cAMP signaling pathway has also been shown to upregulate Bim and induce apoptosis in leukemic and lymphoma cells (Meyers et al., 2009; Huseby et al., 2011; Zambon et al., 2011). cAMP signaling also upregulates Bim in normal T and B lymphocytes, but of note, in contrast to leukemic cells, the upregulation of Bim in normal lymphoid cells in response to cAMP signaling does not lead to apoptosis (Meyers et al., 2009). It was reported that stimulation of cAMP signaling enhanced dexamethasone upregulation of Bim and promoted increased apoptosis of leukemic and lymphoma cells, suggesting that Bim may be a convergence point for the increased response seen when these two signaling pathways are stimulated (Zhang and Insel, 2004). Our results confirm this and also point to increased upregulation of Bim as being a possible convergence point for the synergistic effects of cAMP and glucocorticoid signaling on inducing apoptosis of the glucocorticoid-sensitive CEM-S2 and glucocorticoid-resistant CEM-R8 cells that we employed in this study. However, we did not see evidence for this in multiple myeloma cells. In the glucocorticoid-sensitive MM.1S myeloma cells, although stimulation of the glucocorticoid and cAMP signaling pathways both stimulated upregulation of Bim protein expression, there was no further enhanced expression of Bim protein when both signaling pathways were stimulated together, even though there was enhanced promotion of apoptosis when both signaling pathways were stimulated. And in the glucocorticoid-resistant MM.1R myeloma cell line, no upregulation of Bim protein expression was seen in response to glucocorticoid signaling, cAMP signaling, or stimulation of both signaling pathways together. Inasmuch as cAMP signaling clearly induced apoptosis of MM.1R cells, our findings suggest that a mechanism(s) other than Bim induction must come into play to account for the apoptosis that results in response to cAMP stimulation in these cells.

Studies from the Lerner laboratory had shown that stimulation of the cAMP signaling pathway with rolipram and forskolin led to activation of protein phosphatase 2A, dephosphorylation of Bad on ser112, and translocation of Bad to mitochondria, suggesting that activation of the proapoptotic protein Bad may also be important for apoptosis induced by cAMP signaling (Moon and Lerner, 2003). In this study we saw little upregulation of total Bad protein in either CEM-S2 or CEM-R8 cells, but we saw considerable upregulation of Bad protein in both the MM1.S and MM1.R multiple myeloma cells. Moreover, we observed dephosphorylation of Bad on ser112 in response to stimulation of cAMP signaling in all cell types studied here, CEM-S2, CEM-R8, MM.1S and MM.1R, and this dephosphorylation was greatly potentiated by stimulation of the glucocorticoid pathway concurrent with the cAMP signaling pathway. These results suggest that Bad may be an important mediator of apoptosis in multiple myeloma cells in response to these signaling pathways, and that dephosphorylation and activation of Bad may also be a convergence point for induction of apoptosis by the glucocorticoid and cAMP signaling pathways.

Studies have shown that the ability of glucocorticoids to induce apoptosis of leukemic cells may depend in part on the relative balance of the proapoptotic protein Bim and the antiapoptotic protein Bcl-2 present in the target cell (Ploner et al., 2005; Jing et al., 2015), and the same may be true for Bim and the antiapoptotic protein Mcl-1 in multiple myeloma cells (Gomez-Bougie et al., 2004; Follin-Arbelet et al., 2013). Models put forth by the Letai laboratory describe Bim as an activator protein, capable of binding directly to Bax, resulting in mitochondrial membrane permeabilization, whereas Bad is described as a sensitizer protein capable of binding to antiapoptotic proteins like Bcl-2 and Mcl-1, which thus frees Bim from its tethered association with Bcl-2 or Mcl-1, allowing it to bind Bax and initiate permeabilization of the mitochondrial membrane (Brunelle and Letai, 2009). Cells in which activator proteins such as Bim are in higher abundance may be primed for death by being particularly sensitive to exposure to sensitizer domains provided by Bad, such that an increase in Bad may tip the balance and induce apoptosis. Inasmuch as Bad was induced in MM.1S and MM.1R cells, as well as dephosphorylated on ser112, in response to cAMP signaling, this may in part explain the mechanism whereby cAMP signaling induces apoptosis in multiple myeloma. Small molecule BH3-mimetics that mimic the BH3 binding domain of proapoptotic proteins such as Bad have been found to be effective inducers of apoptosis, and at least one such BH3-mimetic, ABT-199, has been entered into clinical trials for treatment of multiple myeloma, as well as for lymphomas and leukemias (Correia et al., 2015). Our results presented in this study would suggest that stimulation of the cAMP signaling pathway with PDE inhibitors in conjunction with BH3-mimetics may well provide an even more effective therapeutic strategy for treatment of leukemia and multiple myeloma.

Several studies have shown altered expression of PDEs in leukemic patients to be associated with poorer treatment outcomes, often related to glucocorticoid resistance in the treatment of these patients. For example, CLL patients with higher expression levels of PDE7B have a several-year shorter median time-to-treatment compared to patients with lower levels of PDE7B expression (Zhang et al., 2011). Overexpression of PDE4B in patients with diffuse large B cell lymphoma (DLBCL) was associated with relapse after chemotherapy, and overexpression of PDE4B in DLBCL impinged on the same genes that are normally active in glucocorticoid resistance (Smith et al., 2005; Kim et al., 2011). A study of 2,535 children with ALL showed single nucleotide polymorphisms in the PDE4B gene to be associated with a high risk of relapse in children newly diagnosed with ALL, and it was suggested that this may be due to the resultant glucocorticoid resistance that develops due to the PDE4B polymorphisms, which further underscores the importance of targeting PDE4 in overcoming glucocorticoid resistance (Yang et al., 2012). When high throughput screening technology was employed to identify agents that synergize with dexamethasone to inhibit proliferation of MM.1S and DLBCL cells, the compounds identified to produce the greatest amount of synergy were adenosine A2A receptor agonists and PDE 2,3,4, and 7 inhibitors (Rickles et al., 2010). In a study examining the *in vitro* antileukemic activity of 20 different anticancer agents against tumor cells from CLL patients aimed at identifying agents active in poor-prognostic subgroups, it was found that prednisolone and rolipram displayed high CLL specificity and high activity in CLL with unmutated IGHV genes and when prednisolone and rolipram were combined they displayed considerable synergy against these CLL cells, thereby identifying rolipram as an agent with high activity in cells from patients with poor prognosis (Lindhagen et al., 2009). Recent studies from the Lerner laboratory showed that rolipram induced apoptosis of both IGHV unmutated and mutated CLL cells, suggesting that cAMP signaling may abrogate a TLR9-mediated survival signal in prognostically unfavorable IGHV unmutated CLL cells, and indicating that PDE4 inhibitors may well be of clinical utility in CLL or autoimmune diseases that are driven by TLR-mediated signaling (Tan et al., 2015). PDE inhibitors are currently under intense development for a wide range of illnesses and are rapidly being approved for clinical use (Houslay et al., 2005; Bender and Beavo, 2006; Lugnier, 2006; Epstein, 2012; Maurice et al., 2014; Ahmad et al., 2015). Of note, two PDE4 inhibitors, roflumilast and apremilast, are already approved and in clinical use for the indications of chronic obstructive pulmonary disease and psoriatic arthritis, respectively. These studies, as well as the results of our study presented here, suggest that PDE inhibitors may provide valuable tools for overcoming glucocorticoid resistance and thereby improve the treatment outcome of patients with lymphomas, leukemias, and multiple myeloma. As such, clinical studies should clearly be undertaken with PDE inhibitors, either as single agents, or in combination with BH3-mimetics and/or established therapeutic agents for treatment of patients with leukemia, lymphoma and multiple myeloma in relation to glucocorticoid resistance.

## Conflict of Interest Statement

The authors declare that the research was conducted in the absence of any commercial or financial relationships that could be construed as a potential conflict of interest.
